# Comprehensive Frailty Assessment in Chronic Kidney Disease

**DOI:** 10.1093/geroni/igaf122.2366

**Published:** 2025-12-31

**Authors:** Danya Pradeep Kumar, Nicole Oblon, Jacob Hiss, Mary Piedad, Kate Young, Aditi Gupta

**Affiliations:** University of Kansas Medical Center, Kansas City, Kansas, United States; University of Kansas Medical Center, Kansas City, Kansas, United States; University of Kansas Medical Center, Kansas City, Kansas, United States; Kansas City University, Kansas City, Kansas, United States; University of Kansas Medical Center, Kansas City, Kansas, United States; University of Kansas Medical Center, Kansas City, Kansas, United States

## Abstract

Individuals with chronic kidney disease (CKD) are at a high risk of frailty, a multidimensional geriatric syndrome of physiological decline. The ideal method to assess frailty in individuals with CKD is unknown. In this single center cross-sectional study of individuals with CKD stage 3-5, we compared frailty as assessed by Fried’s Frailty Phenotype (FFP) and the Comprehensive Frailty Index (CFI) in individuals with CKD. FFP is comprised of 5 criteria – slowness, weakness, low-activity, exhaustion and weight-loss. CFI ranges from 0-1 and was calculated using 50 health deficits from various health domains based on standardized procedures. Forty-nine individuals (59% female; age: 68.8 ± 8.86 years; eGFR: 34.8 ± 8.53 mL/min/1.73m2) with CKD completed the assessments. With FFP, 20, 25, and 4 participants were non-frail, pre-frail, and frail respectively. With the CFI, 16, 23, and 10 participants were non-frail, pre-frail, and frail respectively. There was a discrepancy in frailty status by FFP vs. CFI in 26 participants (53%). Domain-wise comparison also showed a discrepancy between FFP and CFI. Of the 49 participants, 39 (80%) had discrepancy in exhaustion, 22 (45%) in low-activity, and 6 (12%) in slowness. There were no direct comparison for weakness or weight loss. These data show that frailty status for an individual can differ depending on the frailty assessment used. Compared to FFP, CFI may provide more details on the cause of frailty. Clinical frailty assessments need to be refined to identify suitability of different interventions in managing frailty.

